# Methylation of DACT2 promotes breast cancer development by activating Wnt signaling

**DOI:** 10.1038/s41598-017-03647-3

**Published:** 2017-06-12

**Authors:** Jingyi Li, Meiying Zhang, Tao He, Hongxia Li, Tingting Cao, Lili Zheng, Mingzhou Guo

**Affiliations:** 1 Department of Gastroenterology and Hepatology, Chinese PLA General Hospital, Beijing, 100853 China; 2 Medical College of NanKai University, Tianjin, 300071 China; 3 Colloge of Life Science and Bioengineering, Beijing University of Technology, 100124 Beijing, China; 4 Department of Endocrinology, the First Affiliated Hospital, Zhengzhou University, Zhengzhou, 450052 China

## Abstract

Breast cancer is the most common malignant tumor in women worldwide. To explore the role of DACT2 in breast cancer, 5 cell lines and 153 cases of primary cancer were studied. The expression of DACT2 was detected in BT474, MDA-MB-231 and BT549 cells, while no expression was found in MDA-MB-468 and HBL100 cells. Complete methylation of DACT2 was found in MDA-MB-468 and HBL100 cells, partial methylation was observed in BT474 and BT549 cells, and no methylation was detected in MDA-MB-231 cells. Restoration of DACT2 expression was induced by 5-Aza in MDA-MB-468 and HBL100 cells. DACT2 was methylated in 49.7% (76/153) of primary breast cancer samples. Methylation of DACT2 was significantly associated with tumor size (*P* < 0.05). Reduced DACT2 expression was significantly associated with promoter region methylation in primary breast cancer (*P* < 0.05). DACT2 suppressed breast cancer cell growth and induced G1/S phase arrest in breast cancer cells. DACT2 inhibited Wnt/β-catenin signaling in human breast cancer cells and suppressed breast cancer cell tumor growth in xenograft mice. In conclusion, our results demonstrate that DACT2 is frequently methylated in human breast cancer, methylation of DACT2 activates Wnt signaling, and DACT2 suppresses breast cancer cell growth both *in vitro* and *in vivo*.

## Introduction

Breast cancer is the most common malignancy in women worldwide, and it is the second leading cause of cancer related death among women^1^. The Wnt signaling pathway plays an important role in cell proliferation and differentiation and its constitutive activation has been implicated in tumorigenesis of different cancer types^2^. Activation of the Wnt signaling pathway is associated with breast tumorigenesis and poor prognosis^3, 4^. APC and β-catenin are major components of Wnt signaling pathway. In contrast to colorectal cancer, mutations in APC and β-catenin are rare in human breast cancer^5, 6^. Aberrant epigenetic changes have been reported as important events in breast cancer development^7, 8^. Thus, exploring novel epigenetic markers of breast cancer may contribute to the early detection, prognosis and development of targeted therapy for this disease.

Dapper, a Dishevelled-associated antagonist of β-catenin (DACT), was reported to interact with Dishevelled, a key component of Wnt signaling^9^. Human DACT1 and DACT2 were identified and characterized by Katoh *et al*. in 2003^10^. Human DACT2 is located on chromosome 6q27, a location in which there is frequent loss of heterozygosity (LOH) in human cancers^11^. DACT2 gene has been reported to be frequently methylated in human lung, liver, gastric, colon, thyroid and esophageal cancers. These reports indicate that DACT2 expression is silenced and Wnt signaling is activated by DACT2 promoter region methylation^11–15, 22^. In this study, we explored the epigenetic regulation and function of DACT2 in human breast cancer.

## Results

### DACT2 is silenced by promoter region hypermethylation in breast cancer cells

**Figure 1:**
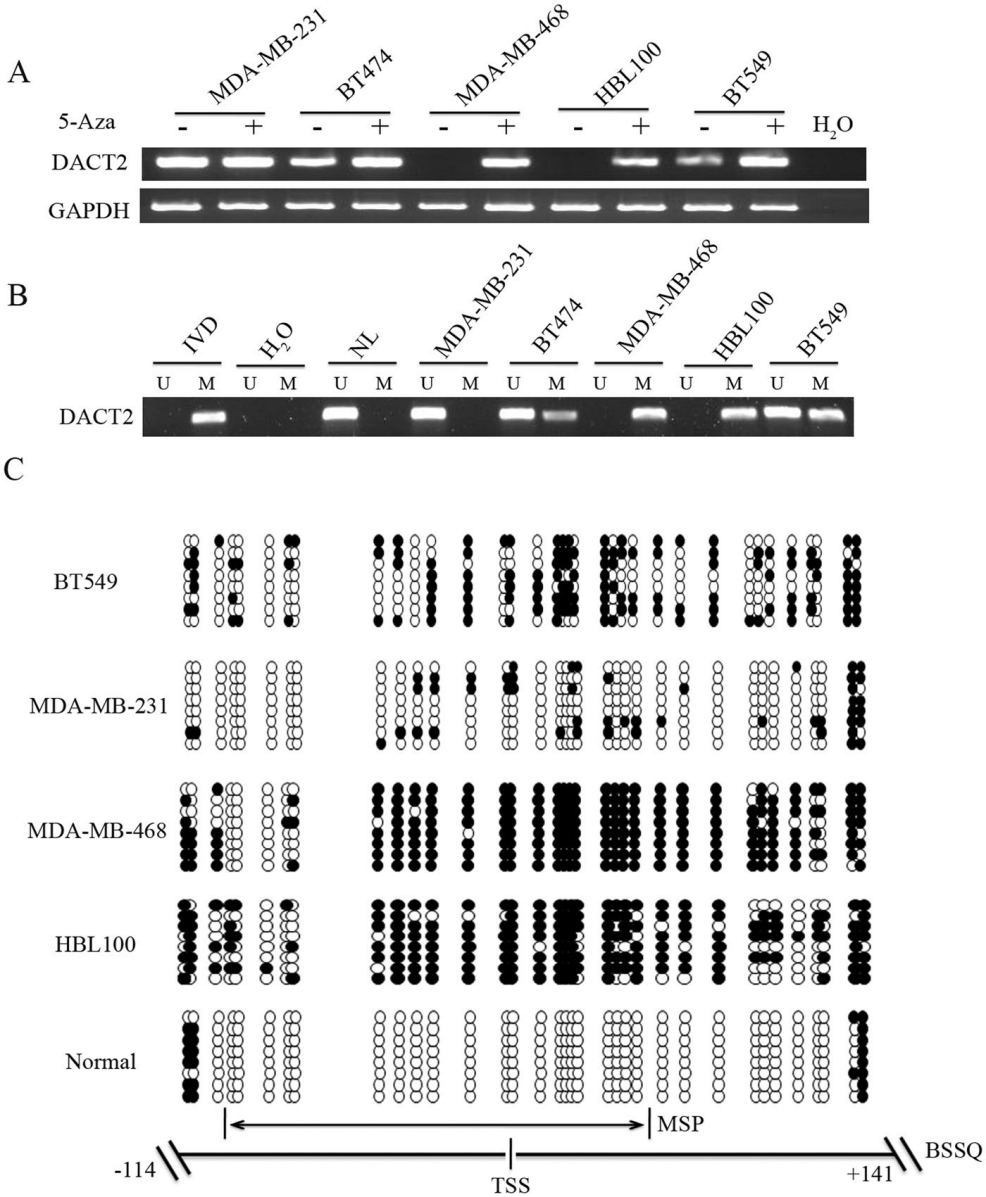
Expression of DACT2 was silenced by DNA methylation in breast cancer cell lines (**A**) RT-PCR shows the levels of DACT2 expression. MDA-MB-231, BT474, MDA-MB-468, HBL100 and BT549 are breast cancer cell lines. (−) untreated; (+) 5-Aza treated; H_2_O: double distilled water. GAPDH was used as an internal control. (**B**) MSP results of DACT2 in breast cancer cell lines. IVD: *in vitro* methylated DNA; NL: normal lymphocyte DNA; M: methylated alleles; U: unmethylated alleles. (**C**) Bisulfite sequencing results: Double-headed arrow indicates the region of the MSP product. Filled circles: methylated CpG sites; open circles: unmethylated CpG sites. TSS: transcription start site.

To explore the regulation of DACT2 gene in breast cancer, the expression of DACT2 was examined in breast cancer cell lines using semi-quantitative RT-PCR. The expression of DACT2 was detected in BT474, MDA-MB-231 and BT549 cells, and no DACT2 expression was found in MDA-MB-468 and HBL100 cells (Fig. 1A). DACT2 promoter region methylation was examined by methylation-specific PCR (MSP). Complete methylation of DACT2 was found in MDA-MB-468 and HBL100 cells, partial methylation was observed in BT474and BT549 cells, and no methylation was detected in MDA-MB-231 cells (Fig. 1B). These results indicate that DACT2 was silenced by promoter region methylation.

To further validate that the expression of DACT2 was regulated by promoter region methylation, breast cancer cell lines were treated with 5-Aza. Re-expression of DACT2 was found in MDA-MB-468 and HBL100 cell lines after 5-Aza treatment. Increased expression of DACT2 was induced by 5-Aza in BT474 and BT549 cell lines. No expression changes in DACT2 were found in MDA-MB-231 cells before and after 5-Aza treatment (Fig. 1A). These results demonstrate that the expression of DACT2 is regulated by promoter region methylation. To validate the efficiency of the MSP primers, bisulfite sequencing was employed. Dense methylation was observed in the promoter region of DACT2 in MDA-MB-468 and HBL100 cells, and unmethylation was found in MDA-MB-231 cells and normal breast tissue samples. Partial methylation was found in BT549 cells (Fig. 1C).

### DACT2 is frequently methylated in human breast cancer

**Figure 2:**
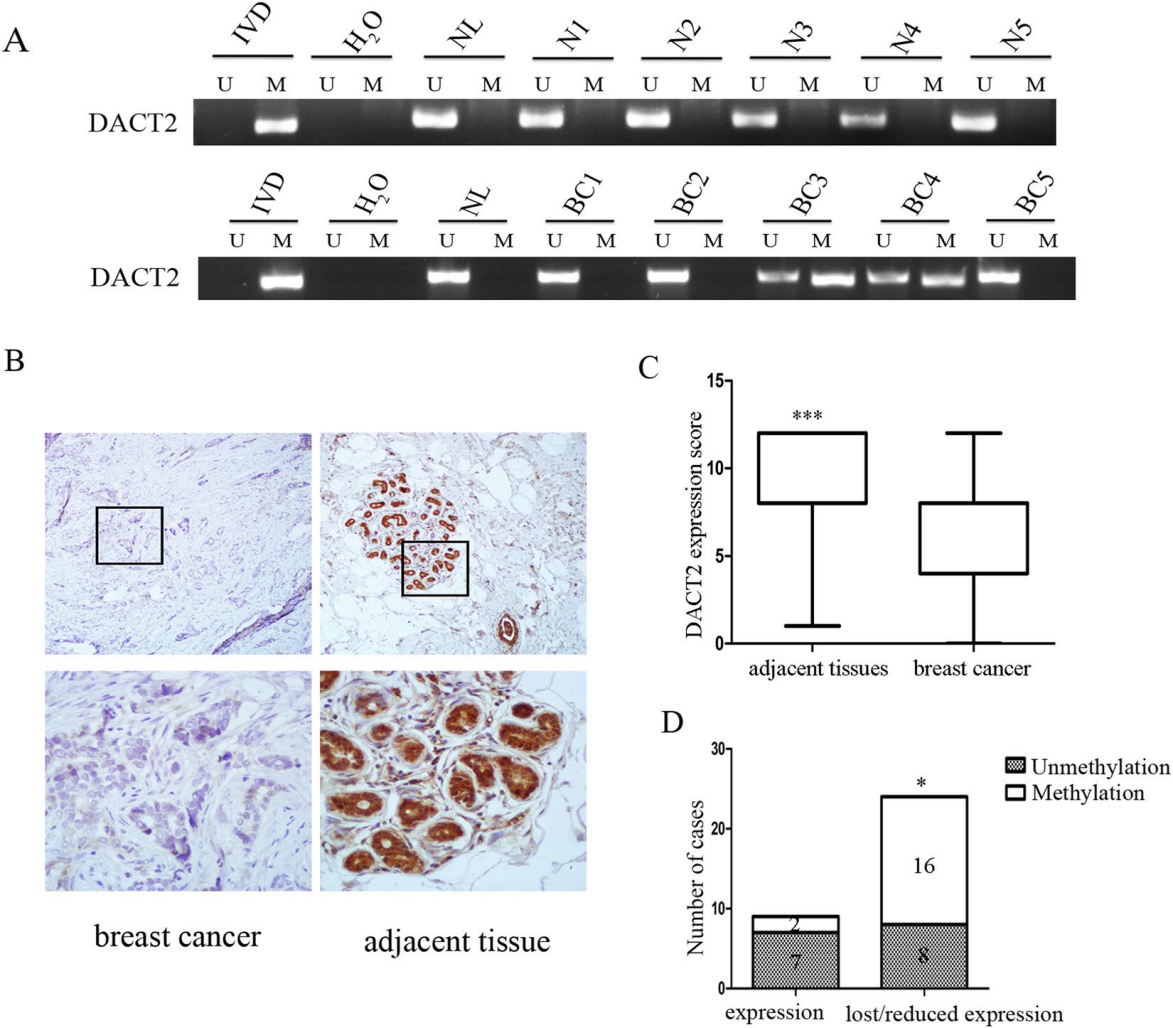
Representative MSP and IHC results of DACT2 in human primary breast cancer (**A**) Representative MSP results of DACT2 in normal breast tissues (N1, N2, N3, N4 and N5) and primary breast cancer tissues (BC). (**B**) Representative IHC staining of DACT2 in breast cancer (left panels) and adjacent tissue (right panels). Upper panels: 100×; lower panels: 400×. (**C**) DACT2 expression scores are shown as box plots. The bottom and top of the boxes represent the 25th and 75th percentiles, respectively; vertical bars represent the range of data. The levels of expression are higher in adjacent tissue compared to cancer samples. ***P < 0.001 (**D**) The levels of DACT2 expression and DNA methylation status are shown as bar diagram. Reduced expression of DACT2 was significantly associated with promoter region hypermethylation. *P < 0.05.

Methylation of DACT2 was examined in 153 cases of human primary breast cancer and 5 cases of normal breast tissue samples (Fig. 2A). DACT2 was methylated in 49.7% (76/153) of human primary breast cancer, and no methylation was found in normal breast tissue samples. Methylation of DACT2 was significantly associated with tumor size (*P* < 0.05, Table 1). No association was found between DACT2 methylation and age, tumor grade, tumor stage, lymph node metastasis and the expression of progesterone receptor (PR), estrogen receptor (ER) or Human epidermal growth factor receptor 2 (HER2) (all *P* > 0.05).

**Table 1:** The association of DACT2 methylation status and clinical factors in human breast cancer patients.

		Methylation status		
Clinical parameter	Number (n = 153)	Unmethylated N = 77(50.3%)	Methylated N = 76(49.7%)	*p* value
Age				
<50	72	38	34	0.57
≥50	81	39	42	
Tumor grade				
I–II	93	44	49	0.35
III	60	33	27	
Tumor stage				
1–2	121	63	58	0.40
3–4	32	14	18	
Tumor size				
<4 cm	114	63	51	0.037*
≥4 cm	39	14	25	
Lymph node metastasis				
no	79	44	35	0.17
yes	74	33	41	
ER status				
Positive	30	15	15	0.97
Negative	123	62	61	
PR status				
Positive	48	23	25	0.69
Negative	105	54	51	
HER2 status				
Positive	131	68	63	0.57
Negative	22	9	13	

^*^ *P* values are obtained from *χ* ^2^ test, significant difference, *P < 0.05.

The expression of DACT2 was analyzed by immunohistochemistry in 33 cases of available matched breast cancer and adjacent tissue samples. DACT2 staining was found mainly in cytoplasm (Fig. 2B). The levels of DACT2 expression were significantly lower in cancer tissues compared to adjacent normal tissue samples (Fig. 2C, *P* < 0.001). Low level expression of DACT2 was found in 24 cases of cancer samples, 16 of which were methylated. Reduced DACT2 expression was significantly associated with promoter region hypermethylation (Fig. 2D, *P* < 0.05). These results further suggest that the expression of DACT2 is regulated by promoter region methylation in breast cancer.

### Restoration of DACT2 expression suppresses cell growth in human breast cancer cells

**Figure 3:**
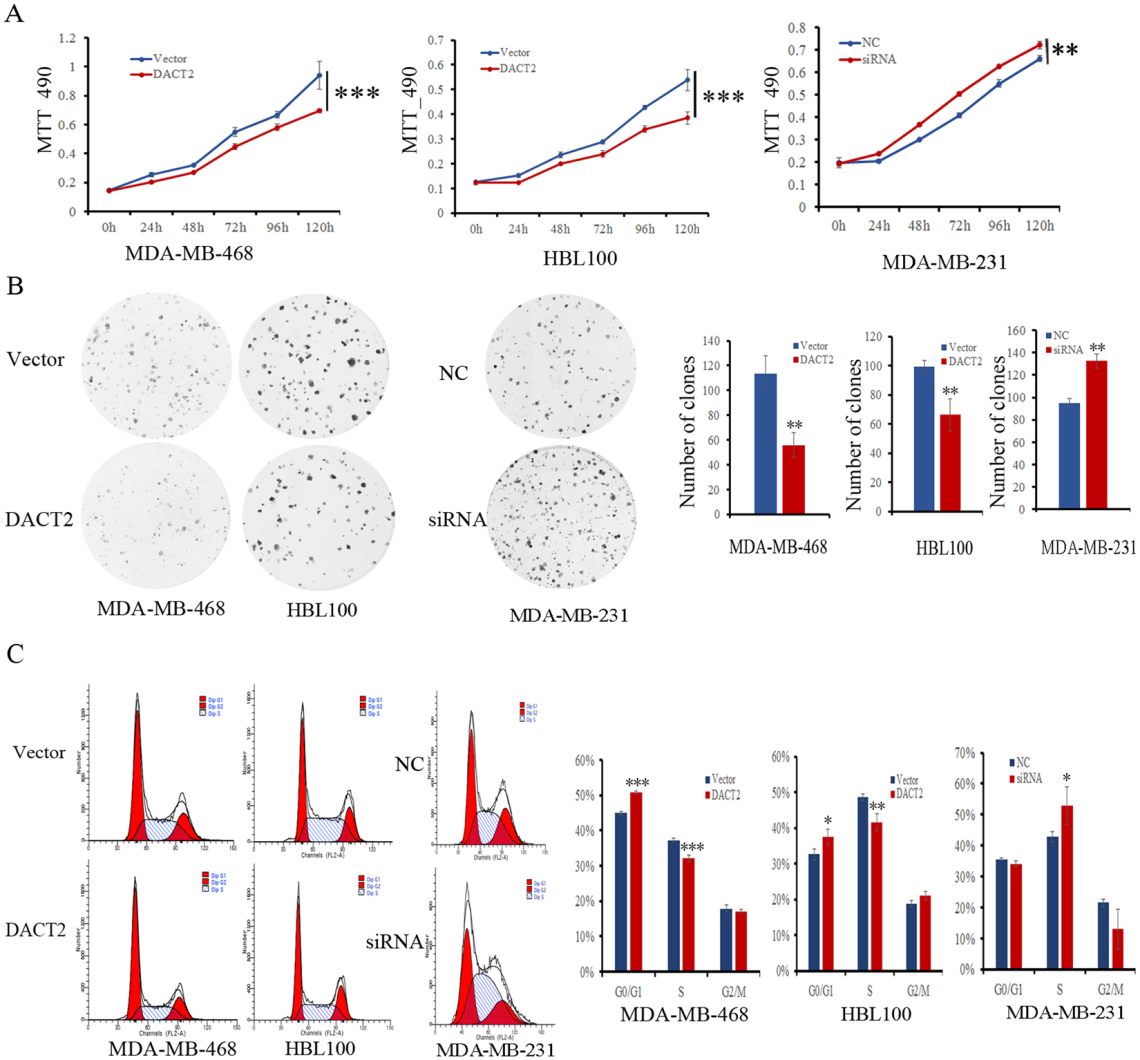
The effects of DACT2 on breast cancer cell proliferation and cell cycle (**A**) The effects of *DACT2* on cell proliferation were measured by the MTT assay for 120 hours. Each experiment was repeated three times. Vector: control group; DACT2: DACT2 re-expressed breast cancer cells; NC: negative control; siRNA: knockdown DACT2 breast cancer cells. **P < 0.01, ***P < 0.001. (**B**) The effects of DACT2 on colony formation in breast cancer cell lines. Each experiment was repeated three times. **P < 0.01 (**C**) Cell phase distribution in DACT2 unexpressed and re-expressed MDA-MB-468 and HBL100 cells, as well as cell phase distribution before and after knockdown of DACT2 in MDA-MB-231 cells. Each experiment was repeated three times. *P < 0.05, **P < 0.01, ***P < 0.001.

To evaluate the effects of DACT2 on breast cancer cell proliferation, the MTT assay was employed in MDA-MB-468 and HBL100 cells. The OD values were 0.94 ± 0.10 *vs*. 0.69 ± 0.01 (*P* < 0.001) in MDA-MB-468 cells and 0.54 ± 0.04 *vs*. 0.38 ± 0.02 (*P* < 0.001) in HBL100 cells before and after restoration of DACT2 expression. The effect of DACT2 on cell growth was further validated by knocking down DACT2 in MDA-MB-231 cells. The OD values were 0.66 ± 0.02 *vs*. 0.72 ± 0.01 (*P* < 0.01) before and after knockdown DACT2 in MDA-MB-231 cells (Fig. 3A).

Colony formation assay was then performed in MDA-MB-468 and HBL100 cells. The colony numbers were 113.7 ± 14.4 *vs*. 55.7 ± 10.1 (*P* < 0.01) in MDA-MB-468 cells and 99.3 ± 4.0 *vs*. 66.3 ± 11.2 (*P* < 0.01) in HBL100 cells before and after re-expression of DACT2. The effect of DACT2 on clonogenicity was further validated by knocking down DACT2 in MDA-MB-231 cells. The clone number was 94.7 ± 4.2 *vs*. 132.7 ± 6.4 (*P* *<* 0.01) before and after knockdown of DACT2 in MDA-MB-231 cells (Fig. 3B). These results suggest that DACT2 suppresses breast cancer cell growth.

### DACT2 induces G1/S checkpoint arrest in human breast cancer cells

To analyze the effects of DACT2 on cell cycle, flow cytometry assay was performed. The cell phase distributions in DACT2 unexpressed and re-expressed MDA-MB-468 cell lines were 44.90 ± 0.56% *vs*. 50.75 ± 0.42% in G0/G1 phase, 37.26 ± 0.49% *vs*. 32.12 ± 0.83% in S phase, and 17.84 ± 0.99% *vs*. 17.13 ± 0.41% in G2/M phase (Fig. 3C). The percentage of cells in S phase was reduced significantly (*P* < 0.001), and the percentage of cells in G0/G1 phase was increased significantly (*P* < 0.001) after restoration of DACT2 expression. In HBL100 cells, the cell phase distributions were 32.58 ± 1.48 *vs*. 37.48 ± 2.17% in G0/G1 phase, 48.63 ± 0.83 *vs*. 41.51 ± 2.4% in S phase, and 18.79 ± 0.97  *vs*. 21.01 ± 1.18% in G2/M phase before and after restoration of DACT2 expression (Fig. 3C). The percentage of S phase cells was reduced significantly (*P* < 0.01), and the ratio of G0/G1 phase cells was increased significantly after re-expression of DACT2 in HBL100 cells (*P* < 0.05). The effect of DACT2 on cell cycle was further validated by knocking down DACT2 in DACT2 highly expressed MDA-MB-231 cells. The distribution of cell phases was 35.57 ± 0.37% *vs*. 34.13 ± 0.92% in G0/G1 phase, 42.87 ± 1.51% *vs*. 52.90 ± 6.03% in S phase, and 21.56 ± 1.15% *vs*. 12.98 ± 6.45% in G2/M phase. The S phase was significantly increased after knockdown DACT2 in MDA-MB-231 cells (*P* < 0.05, Fig. 3C). These results suggest that DACT2 induced G1/S arrest in breast cancer cells.

The effects of DACT2 on cell cycle were further validated by evaluating the expression levels of cyclin D1 and cyclin E1. Under western blot detection, the expression levels of cyclin D1 and cyclin E1 were reduced after re-expression of DACT2 in MDA-MB-468 and HBL100 cells. The expression levels of cyclin D1 and cyclinE1 were increased after knock down of DACT2 in MDA-MB-231 cells (Fig. 5A). These results further demonstrate that DACT2 induced the G1/S checkpoint arrest in breast cancer cells.

### DACT2 suppresses cell migration and invasion in breast cancer cells

**Figure 4:**
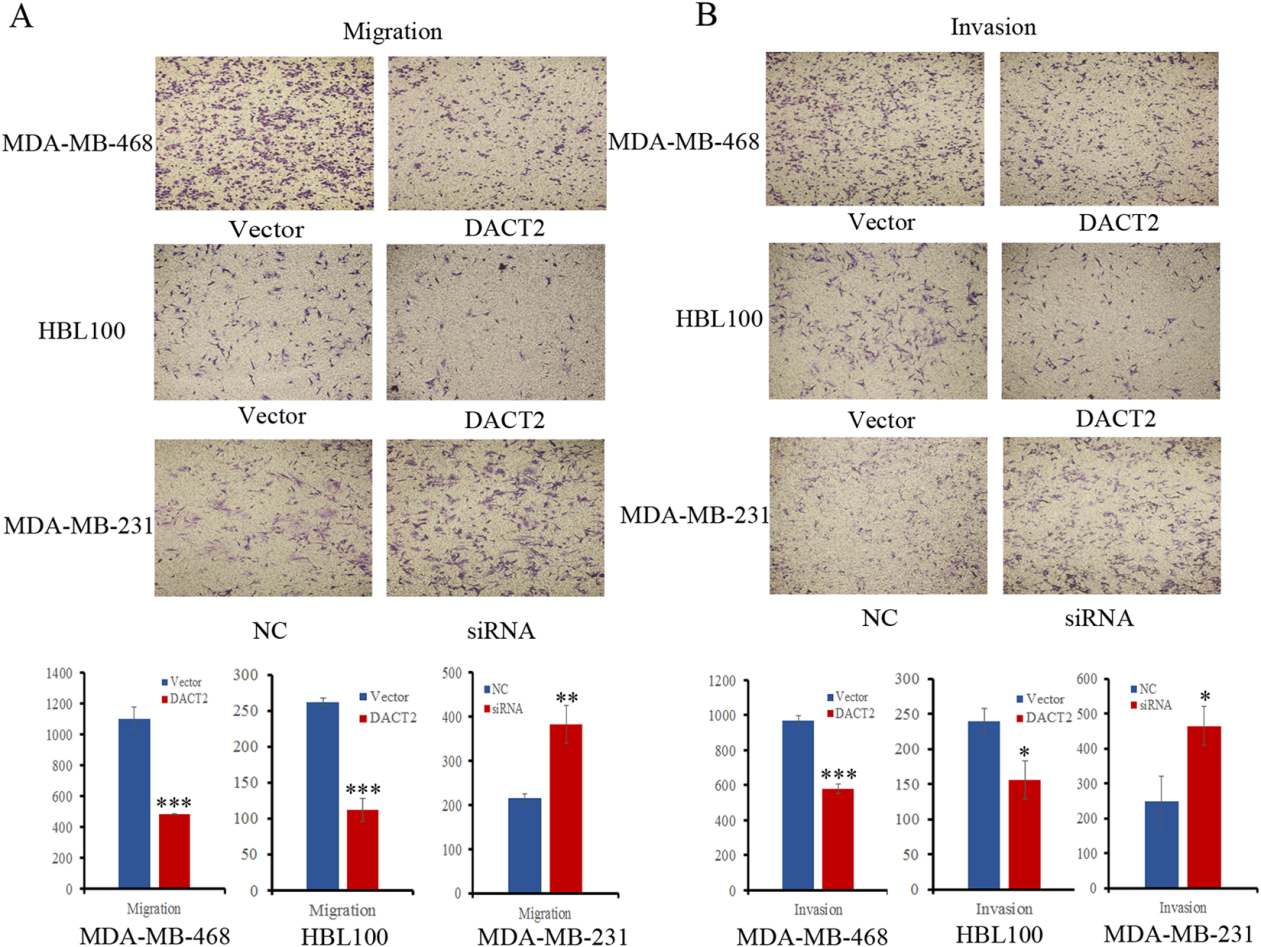
The effects of DACT2 on breast cancer cell migration and invasion (**A**) Transwell results show cell migration in DACT2 unexpressed and re-expressed MDA-MB-468 and HBL100 cells, as well as migration of MDA-MB-231 cells before and after knockdown of DACT2. Each experiment was repeated three times. **P < 0.01, ***P < 0.001 (**B**) Transwell results show cell invasion in DACT2 unexpressed and re-expressed MDA-MB-468 and HBL100 cells, as well as invasion of MDA-MB-231 cells before and after knockdown of DACT2. Each experiment was repeated three times. *P < 0.05, ***P < 0.001.

To evaluate the effects of DACT2 on cell migration and invasion, the transwell assays were used. The number of migratory cells was 1102.33 ± 76.57 *vs*. 483.00 ± 3.61 for MDA-MB-468 cells and 262.00 ± 5.00 *vs*. 112.00 ± 16.00 for HBL100 cells before and after restoration of DACT2 expression. The cell number was reduced significantly after re-expression of DACT2 in MDA-MB-468 and HBL100 cells (both *P* < 0.001, Fig. 4A). The number of migratory cells was 216.00 ± 9.85 *vs*. 382.33 ± 42.12 before and after knockdown of DACT2 in MDA-MB-231 cells. The cell number was increased significantly after knockdown of DACT2 in MDA-MB-231 cells (*P* < 0.01, Fig. 4A). The number of invasive cells was 966.67 ± 30.41 *vs*. 578.67 ± 27.47 in MDA-MB-468 cells and 239.67 ± 17.79 *vs*. 156.00 ± 27.62 in HBL100 cells before and after restoration of DACT2 expression. The invasive cell number was reduced significantly after re-expression of DACT2 in MDA-MB-468 and HBL100 cells (*P* < 0.001, *P* < 0.05, respectively, Fig. 4B). The number of invasive cells was 247.33 ± 72.22 *vs*. 463.67 ± 55.41 before and after knockdown of DACT2 in MDA-MB-231 cells. The invasive cell number was increased significantly after knockdown of DACT2 in MDA-MB-231 cells (*P* < 0.05, Fig. 4B). These results suggest that DACT2 suppresses breast cancer cell migration and invasion.

### DACT2 is a Wnt/β-catenin signaling pathway inhibitor in human breast cancer

**Figure 5:**
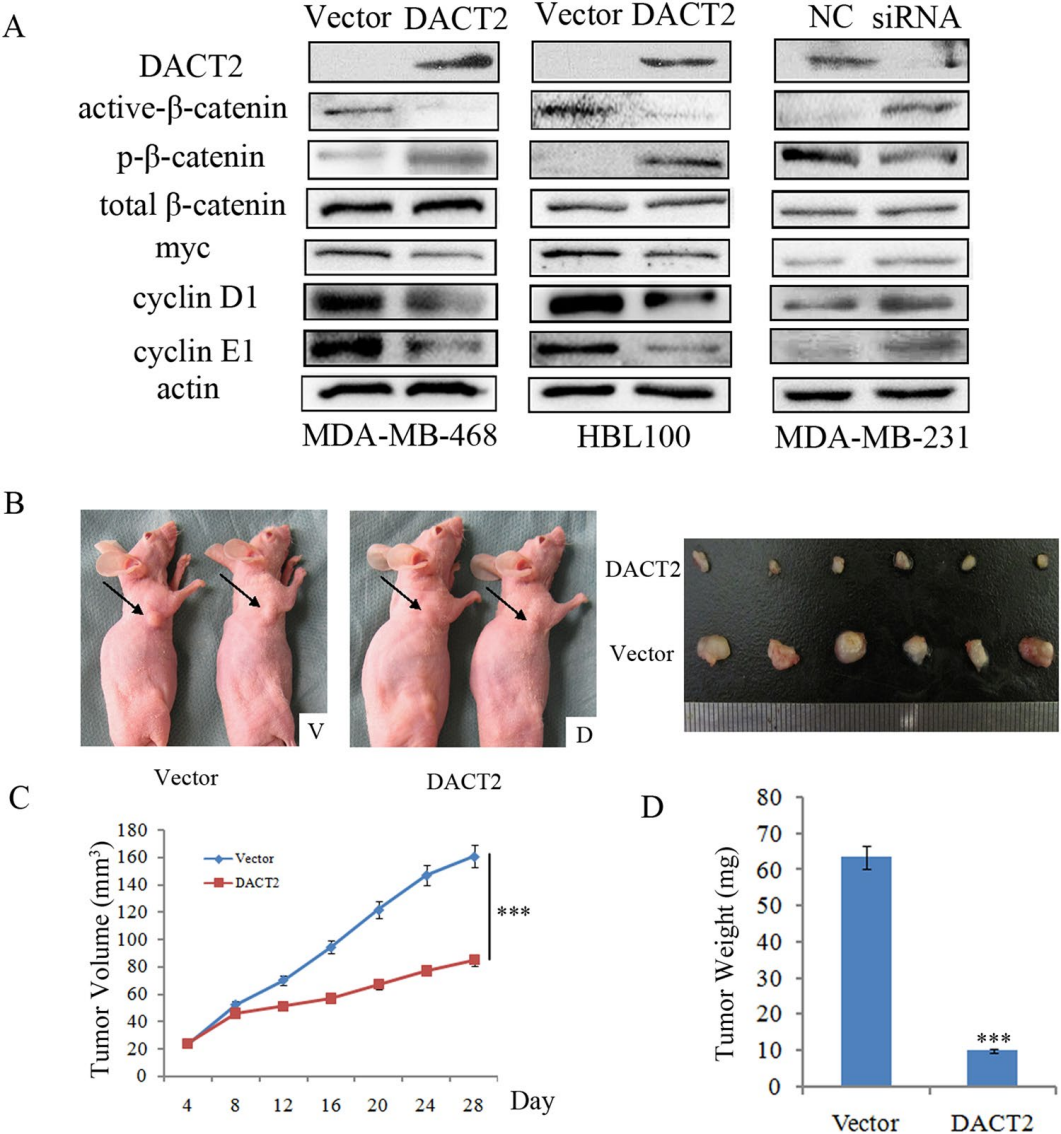
The role of DACT2 in Wnt/β-catenin signaling and the effects of DACT2 on breast cancer cell line xenografts (**A**) Active-β-catenin, p-β-catenin, total β-catenin, myc, cyclin D1 and cyclin E1 were detected by western blot in DACT2 unexpressed (vector) and re-expressed (DACT2) MDA-MB-468 and HBL100 cells. The results were validated by knocking down DACT2 in MDA-MB-231 cells. (**B**) Representative results of DACT2 re-expressed and unexpressed MDA-MB-468 cell xenografts in mice. V: control group; D: DACT2 re-expressed breast cancer cells group (**C**) Tumor growth curves of DACT2 re-expressed and unexpressed MDA-MB-468 cell xenografts. ***P < 0.001 (**D**) The average weights of DACT2 re-expressed and unexpressed MDA-MB-468 cell xenografts. ***P < 0.001

DACT2 was found to be involved in Wnt/β-catenin signaling in different cancers in our previous reports^11–15^. The mechanism of DACT2 in human breast cancer remains unclear. The effects of DACT2 on Wnt/β-catenin signaling were analyzed by DACT2 overexpression and siRNA knockdown techniques in human breast cancer cells. The levels of non-phospho (active) β-Catenin were reduced and the levels of p-β-catenin were increased while the total level of β-catenin was not changed after re-expression of DACT2 in MDA-MB-468 and HBL100 cells. The levels of downstream target genes, myc and cyclinD1, were reduced after restoration of DACT2 expression (Fig. 5A). The levels of active-β-catenin, myc and cyclinD1 increased, the levels of p-β-catenin decreased, and the levels of total β-catenin did not change after knockdown of DACT2 in MDA-MB-231 cells (Fig. 5A). These results suggest that DACT2 inhibits the Wnt/β-catenin signaling pathway in human breast cancer cells.

### DACT2 inhibits breast cancer cell growth *in vivo*

To further validate the effects of DACT2 on breast cancer cell growth, DACT2 unexpressed and re-expressed MDA-MB-468 cell xenograft mouse models were employed (Fig. 5B). The tumor volumes were 160.7 ± 12.8 mm^3^ and 85.0 ± 4.9 mm^3^ in DACT2 unexpressed and re-expressed MDA-MB-468 cell xenograft mice, respectively. The tumor volumes were significantly smaller in DACT2 re-expressed MDA-MB-468 cell xenograft mice compared to the DACT2 unexpressed MDA-MB-468 cell xenograft mice (*P* < 0.001 Fig. 5C). The tumor weights were 63.7 ± 8.1 mg and 10 ± 2.6 mg in DACT2 unexpressed and re-expressed MDA-MB-468 cell xenograft mice, respectively. The tumor weights were significantly smaller in DACT2 re-expressed MDA-MB-468 cell xenograft mice compared to DACT2 unexpressed MDA-MB-468 cell xenograft mice (*P* < 0.001 Fig. 5D). These results demonstrate that DACT2 suppresses breast cancer cell growth *in vivo*.

## Discussion

The connection of wnt signaling and human cancer was first reported by Kinzler *et al*. in 1991^16^.The DACT family of scaffold proteins was discovered by virtue of their binding to Dvl proteins, central to Wnt and Planar Cell Polarity (PCP) signaling^9^. In zebrafish, DACT1 has a greater impact on β-catenin-dependent signaling and DACT2 has a greater impact on the β-catenin-independent process called planar cell polarity/convergent-extension signaling^17^. DACT3 has been reported to be a negative regulator of canonical Wnt signaling both in mice development and human colorectal cancer^18, 19^. A recent study suggests that there are two Dact3 paralogs (dact3a and dact3b) in zebrafish. The dact3a and dact3b paralogs have not been well studied in development and human diseases. A novel member of DACT gene family, DACT4, was identified in zebrafish in a recent report^20, 21^. The importance of these DACT members in development has been gradually recognized. DACT2 is the best studied DACT member in human diseases^11–15, 22^.

In this study, we found that DACT2 is frequently methylated in human breast cancer, and the expression of DACT2 is regulated by promoter region methylation. Re-expression of DACT2 suppresses breast cancer cell growth *in vitro* and *in vivo*. We demonstrated that DACT2 suppresses human breast cancer growth by inhibiting the Wnt/β-catenin signaling pathway. DACT2 methylation is associated with tumor size. Recently, Xiang *et al*. found that DACT2 inhibits EMT by antagonizing wnt signaling. They also found that DACT2 suppresses breast cancer cell migration and invasion by inducing actin cytoskeleton regorganization^23^. In this study, we also found that DACT2 suppresses breast cancer cell migration and invasion. In conclusion, our data indicate that DACT2 is frequency methylated in human breast cancer and the expression of DACT2 is regulated by promoter region methylation. DACT2 suppresses breast cancer development by inhibiting canonical Wnt signaling. Therefore, DACT2 methylation is a potential breast cancer detection marker.

## Materials and Methods

### Cell Lines and Human Tissue samples

Primary breast cancer samples (153 cases) were collected at the Chinese PLA General Hospital and the First Affiliated Hospital of Zhengzhou University, and tumors were staged according to the American Joint Committee on Cancer (AJCC) Cancer Staging Manual, 2010 (7th edition). All cases of breast cancer were classified by TNM stage, including 25 cases of stage І, 96 cases of stage П, 30 cases of stage Ш and 2 cases of stage IV. The median age of the cancer patients was 52 years old (range 27–82). Five cases of normal breast tissue were collected from non-cancerous patients in the Chinese PLA General Hospital and stored as fresh frozen samples. Thirty-three cases of paraffin blocks were available with matched adjacent tissue samples. All samples were collected following the guidelines approved by the institutional review board of the Chinese PLA General Hospital and the First Affiliated Hospital of Zhengzhou University and with written informed consent from patients. Five breast cancer cell lines (MDA-MB-231, BT474, MDA-MB-468, HBL100 and BT549) were previously established from primary breast cancer and maintained in RPMI-1640 (Invitrogen, Carlsbad, CA, USA) supplemented with 10% fetal bovine serum (Hyclone, Logan, UT).

### 5-Aza-2’-deoxycytidine (5-Aza) treatment

Breast cancer cell lines were split to low density (30% confluence) 12 hours before treatment. Cells were treated with 5-Aza-2′-deoxycytidine (Sigma, St. Louis, MO) at a concentration of 2 µM in the growth medium. The growth medium was exchanged every 24 hours for a total of 96 hours treatment.

### RNA Isolation and Semi-quantitative RT-PCR

Total RNA was extracted using Trizol Reagent (Life Technology, MD, USA). Agarose gel electrophoresis and spectrophotometric analysis were used to detect RNA quality and quantity. First strand cDNA was synthesized according to manufacturer’s instructions (Invitrogen, Carlsbad, CA). A total of 5 µg RNA was used to synthesize first strand cDNA. The reaction mixture was diluted to 100 µl with water, then 2.5 µl of diluted cDNA was used for 25 µl PCR reaction. The sequences of PCR primers for DACT2 are as follows: 5′-GGC TGA GAC AAC AGG ACA TCG-3′ (F) and 5′-GAC CGT CGC TCA TCT CGT AAAA-3′ (R). RT-PCR was amplified for 35 cycles. GAPDH was amplified for 25 cycles as an internal control. The primer sequences of GAPDH are as follows: 5′-GAC CAC AGT CCA TGC CAT CAC-3′ (F), and 5′-GTC CAC CAC CCT GTT GCT GTA-3′ (R). The amplified PCR products were examined by 1.5% agarose gels.

### Bisulfite Modification, methylation specific PCR (MSP)

Genomic DNA was prepared by the proteinase K method. MSP primers were designed according to genomic sequences around transcription start sites (TSS) and synthesized to detect methylated (M) and unmethylated (U) alleles. MSP primers for DACT2 are as follows: 5′-GCG CGT GTA GAT TTC GTT TTT CGC-3′ (MF); 5′-AAC CCC ACG AAC GAC GCCG-3′ (MR); 5′-TTG GGG TGT GTG TAG ATT TTG TTT TTT GT-3′ (UF); 5′-CCC AAA CCC CAC AAA CAA CAC CA-3′ (UR). The expected sizes of unmethylated and methylated PCR products are 161 bp and 152 bp, respectively. Bisulfite sequencing (BSSQ) was performed as previously described^24^. BSSQ products were amplified by primers flanking the targeted regions including MSP products. BSSQ primers for DACT2 are as follows: 5′-GGG GGA GGT YGY GGT GAT TT-3′ (F); 5′-ACC TAC RAC RAT CCC AAC CC-3′ (R). The expected size of the BSSQ product is 254 bp.

### Immunohistochemistry

Immunohistochemistry (IHC) was performed in primary breast cancer samples and matched adjacent tissue samples. The DACT2 antibody was diluted to 1:500 (Cat: TA306668, OriGene Tech., MD, USA). The procedure was performed as described previously^25^. The staining intensity and extent of the staining area were scored using the German semi-quantitative scoring system: staining intensity of the nucleus, cytoplasm, and/or membrane (no staining = 0; weak staining = 1; moderate staining = 2; strong staining = 3); extent of stained cells (0% = 0, 1–24% = 1, 25–49% = 2, 50–74% = 3, 75–100% = 4). The final immunoreactive score (0 to 12) was determined by multiplying intensity score and the extent of stained cells score.

### Cell viability detection

MDA-MB-468 and HBL100 cells were seeded into 96-well plates at 1 × 10^3^ cells/well, and 1 × 10^3^ cells were plated into 96-well plates before and after knockdown of DACT2 in MDA-MB-231 cells. The cell viability was measured by MTT assay at 0 h, 24 h, 48 h, 72 h, 96 h and 120 h (KeyGENBiotech, Nanjing, China). Absorbance was measured on a microplate reader (Thermo Multiskan MK3, MA, USA) at a wave length of 490 nm.

### Colony Formation Assay

MDA-MB-468 and HBL100 cells were grown in six-well culture plates for 24 hours before transfection. Cells were transfected with empty vector or DACT2 expression vector according to the manufacturer’s instructions (Invitrogen, CA, USA). Cells were diluted and reseeded at 2000 cells/well in six-well culture plates in triplicate 36 hours later. Growth medium conditioned with G418 (Life Technology, MD, USA) at 300 µg/ml was exchanged every 24 hours. MDA-MB-231 cells before and after knockdown of DACT2 were seeded in 6-well plates at a density of 500 cells per well. After 14 days, cells were fixed with 75% ethanol for 30 min and stained with 0.2% crystal violet for visualization and counting.

### Flow cytometry

MDA-MB-468 and HBL100 cells were transfected with empty vector or DACT2 expression vector according to the manufacturer’s instructions (Invitrogen, CA, USA). Cells were fixed with 70% ethanol and treated using the Cell Cycle Detection Kit (KeyGen Biotech, Nanjing, China) 48 hours after transfection. Cells were then detected using a FACS Caliber flow cytometer (BD Biosciences, CA, USA). MDA-MB-231 cells with or without knockdown of DACT2 were analyzed the cell cycle as well. Cell phase distribution was analyzed using the Modfit software (Verity Software House, ME, USA).

### Transwell assay

Migration: 4 × 10^4^ DACT2 unexpressed and re-expressed MDA-MB-468 and 1 × 10^5^ HBL100 cells, were suspended in 200 µl serum-free RPMI 1640 media and added to the upper chamber of 8.0 µm pore size transwell apparatus (COSTAR transwell Corning Incorporated, MA, USA). Cells that migrated to the lower surface of the membrane were stained with crystal violet and counted in three independent high-power fields (×100) after incubating for 12 hours of MDA-MB-468 cells and 48 hours of HBL100 cells. 4 × 10^4^ MDA-MB-231 cells before and after knockdown of DACT2 were added to the upper chamber of 8.0 µm pore size transwell apparatus. Cells were migrated to the lower surface of the membrane after incubating for 20 hours.

Invasion: the top chamber was coated with a layer of extracellular matrix. 8 × 10^4^ MDA-MB-468 cells and 2 × 10^5^ HBL100 cells were seeded to the upper chamber of a transwell apparatus coated with Matrigel (BD Biosciences, CA, USA) and incubated for 12 hours of MDA-MB-468 cells and 48 hours of HBL100 cells. MDA-MB-231 cells (8 × 10^4^) were added to the upper chamber of a transwell apparatus coated with Matrigel before and after knockdown of DACT2. Cells were invaded to the lower membrane surface after incubating for 20 hours. Cells invaded to the lower membrane surface were stained with crystal violet and counted in three independent high-power fields (×100).

### DACT2 knockdown by siRNA

The selected siRNAs targeting DACT2 and RNAi negative control duplex were used in this study. The sequences are as follows: siRNA duplex (sense: 5′-CCA GCU GUC CUG AGU CUA ATT-3′; antisense: 5′-UUA GAC UCA GGA CAG CUG GTT-3′), RNAi negative control duplex (sense: 5′-UUC UCC GAA CGU GUC ACG UTT-3′; antisense: ACG UGA CAC GUU CGG AGA ATT-3′). RNAi oligonucleotide or RNAi negative control duplex (GenePharma Co. Shanghai, China) were transfected into DACT2 highly expressing MDA-MB-231 cells according to the manufacturer’s instructions.

### Breast cancer cell xenograft mouse model

The human full length DACT2 cDNA (GenBank accession number NM_214462) was cloned into the pLenti6-GFP vector^15^. Primers are as follows: 5′-TGA TCA ATG TGG ACG CCG GGC-3′ (F) and 5′-GTC GAC TCA CAC CAT GGT CAT GAC-3′(R). The HEK-293T cell line was maintained in 90% DMEM (Invitrogen, CA, USA) supplemented with 10% fetal bovine serum. DACT2 expression Lentiviral vector was transfected into HEK-293T cells (5 × 10^6^ per 100 mm dish) using Lipofectamine 3000 Reagent (Invitrogen, CA, USA) at a ratio of 1:3 (DNA mass: Lipo mass). Viral supernatant was collected and filtered after 48 hours. Stably expressing DACT2 cells were selected with Blasticidin (Life Technologies, MD, USA) at concentration of 2.5 µg/ml for 2 weeks. Stably transfected MDA-MB-468 cell line with plenti6-GFP vector and plenti6-DACT2 vector (2 × 10^6^ cells each) were injected subcutaneously into the right flank of 4-week-old female nude mice (n = 6 per group). The diameter of tumors was measured every 4 days for 4 weeks starting 4 days after implantation. Tumor volume was calculated using the following formula: tumor volume (mm^3^) = (length) × (width)^2^ × 0.5^26^. All procedures were approved by the Animal Ethics Committee of the Chinese PLA General Hospital. All experiments were performed in accordance with relevant guidelines and regulations.

### Western Blot

Cells were collected 48h after transfection and cell lysates were prepared using ice-cold Tris buffer (20 mmol/L Tris; pH 7.5) containing 137 mmol/L NaCl, 2 mmol/L EDTA, 1% Triton X, 10% glycerol, 50 mmol/L NaF, 1 mmol/L DTT, PMSF, and a protein phosphatases inhibitor (Applygen Tech. Beijing, China). Antibodies were diluted according to manufacturer’s instructions. Primary antibodies are as follows: DACT2 (OriGene Tech. MD, USA), active-β-catenin (Millipore, CA, USA), p-β-catenin (Bioworld Technology, MN, USA), total-β-catenin (Cell Signaling Tech, MA, USA), myc (Proteintech, IL, USA), cyclin D1 (Proteintech, IL, USA), cyclin E1 (Proteintech, IL, USA) and actin (Bioworld Technology, MN, USA).

### Data Analysis

Data was analyzed by SPSS 17.0 (IBM, NY, USA). The student’s t test, the *χ* ^2^ test analysis and Wilcoxon test analysis were employed. All data were presented as means ± standard deviation (SD). Statistical significance was defined as *P* < 0.05 (*).
